# News and Views on Polysialic Acid: From Tumor Progression and Brain Development to Psychiatric Disorders, Neurodegeneration, Myelin Repair and Immunomodulation

**DOI:** 10.3389/fcell.2022.871757

**Published:** 2022-04-04

**Authors:** Hauke Thiesler, Melike Küçükerden, Lina Gretenkort, Iris Röckle, Herbert Hildebrandt

**Affiliations:** Institute of Clinical Biochemistry, Hannover Medical School, Hannover, Germany

**Keywords:** protein glycosylation, schizophrenia, Alzheimer’s disease, interneuron migration, myelin maintenance and remyelination, microglia and macrophage activation, immune balance, sialic acid-binding immunoglobulin-like lectins (Siglecs).

## Abstract

Polysialic acid (polySia) is a sugar homopolymer consisting of at least eight glycosidically linked sialic acid units. It is a posttranslational modification of a limited number of proteins with the neural cell adhesion molecule NCAM being the most prominent. As extensively reviewed before, polySia-NCAM is crucial for brain development and synaptic plasticity but also modulates tumor growth and malignancy. Functions of polySia have been attributed to its polyanionic character, its spatial expansion into the extracellular space, and its modulation of NCAM interactions. In this mini-review, we first summarize briefly, how the modulation of NCAM functions by polySia impacts tumor cell growth and leads to malformations during brain development of polySia-deficient mice, with a focus on how the latter may be linked to altered behaviors in the mouse model and to neurodevelopmental predispositions to psychiatric disorders. We then elaborate on the implications of polySia functions in hippocampal plasticity, learning and memory of mice in light of recently described polySia changes related to altered neurogenesis in the aging human brain and in neurodegenerative disease. Furthermore, we highlight recent progress that extends the range of polySia functions across diverse fields of neurobiology such as cortical interneuron development and connectivity, myelination and myelin repair, or the regulation of microglia activity. We discuss possible common and distinct mechanisms that may underlie these seemingly divergent roles of polySia, and provide prospects for new therapeutic approaches building on our improved understanding of polySia functions.

## Introduction

**FIGURE 1:**
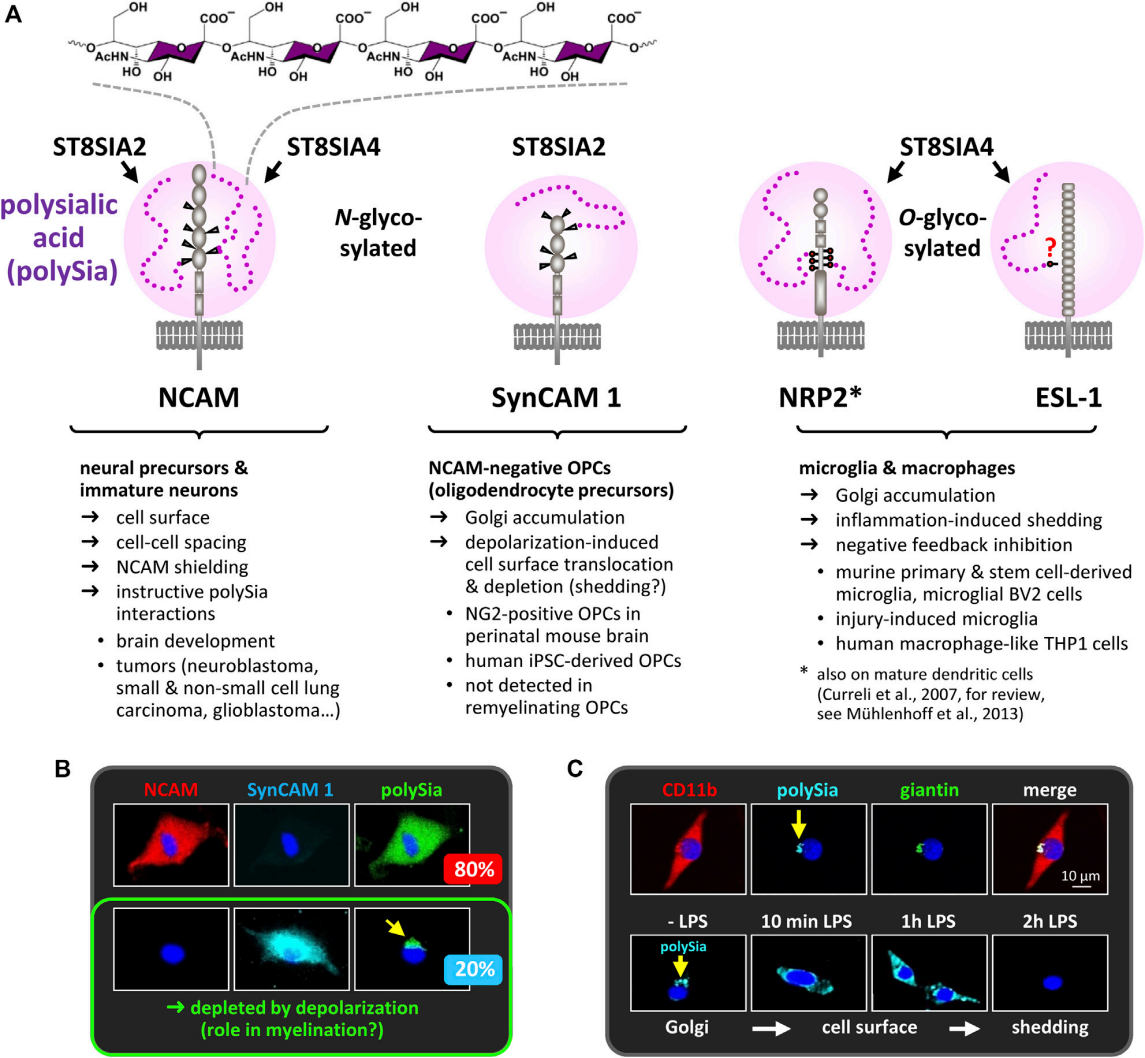
PolySia carriers in the nervous system. **(A)** PolySia structure and schematic representation of polysialylated NCAM, synaptic cell adhesion molecule 1 (SynCAM 1), neuropilin-2 (NRP2) and E-selectin ligand 1 (ESL-1). Involved polySTs, glycosylation sites, type of the core glycan (*N*- or *O*-glycosylation) and selected characteristics are indicated. **(B)** In cultured OPCs, polySia-SynCAM 1 is detected in NCAM-negative OPCs comprising a subpopulation of about 20% of all polySia-positive OPCs. In these cells, polySia-SynCAM 1 accumulates in the Golgi-compartment, but is depleted in response to depolarization (Werneburg et al., 2015a). So far, the possible functions of polySia-SynCAM 1 in OPC differentiation and myelination remain elusive. Notably, polySia-SynCAM 1 was not detected during OPC expansion and remyelination in the cuprizone model (Werneburg et al., 2017; see section “PolySia in myelin maintenance and repair”). **(C)** In cultured microglia (stained with the microglia/macrophage marker CD11b), polySia is also confined to the Golgi compartment, identified by the marker giantin, but rapidly depleted in response to inflammatory activation with bacterial lipopolysaccharide (LPS). See also Figure 2D and section “PolySia in microglia and macrophage activation”. Information on polysialylation of SynCAM 1, NRP2 and ESL-1 is based on Curreli et al., 2007, Galuska et al., 2010, Rollenhagen et al., 2012, 2013, Werneburg et al., 2015a, 2015b, 2016, 2017, and Thiesler et al., 2021. Images in Panels B and C are reproduced from Werneburg et al., 2015a and 2016 with permission from Wiley.

It is now 40 years ago, that α-2,8 glycosidically linked polymers of the sialic acid *N*-acetylneuraminic acid, short polysialic acid (polySia), have been discovered as a unique posttranslational modification of glycoproteins in the early postnatal rat brain (Finne, 1982). This was paralleled by realizing that the remarkable differences between the neural cell adhesion molecule NCAM in embryonic and adult chicken brain are due to a carbohydrate modification that is sensitive to treatment with a bacterial neuraminidase (Rothbard et al., 1982). Since then, the modification of NCAM by polySia and its role during nervous system development and plasticity has been studied in a plethora of papers and extensively reviewed, implementing a major function of polySia as a steric modulator of not only NCAM but also of other cell-cell and cell-matrix interactions, which enables structural plasticity and contrasts with a cell contact stabilizing role of NCAM devoid of polySia (Edelman, 1984; Rutishauser et al., 1988; Rutishauser, 2008). While this is still the prevailing model, deciphering the genetic basis of polysialylation helped to more closely define a role of polySia in controlling NCAM interactions and signaling, and to separate this from polySia functions independent of NCAM modulation. Although broadly reviewed elsewhere (Hildebrandt et al., 2007; Mühlenhoff et al., 2013; Colley et al., 2014; Schnaar et al., 2014), this lays the grounds for more recent studies and therefore is briefly outlined in the following chapter. Although NCAM is by far the major carrier of polySia, a few other polysialylated proteins were detected in the nervous system (Figure 1). One of them is the synaptic cell adhesion molecule SynCAM 1. As detailed in Figure 1, polySia-SynCAM 1 has first been described in the perinatal mouse brain and seems confined to a subset of oligodendrocyte precursor cells (OPCs), the cell population, whose main function is the generation of myelinating oligodendrocytes (Galuska et al., 2010; Werneburg et al., 2015a). While functions of polySia-SynCAM 1 are still elusive, recent progress on the role of polySia on neuropilin-1 (NRP2) and E-selectin ligand 1 (ESL-1) is summarized in the section “PolySia in microglia and macrophage activation”. Concerning the long-standing role of polySia in synaptic transmission, plasticity, learning and memory, however, the reader is referred to other expert reviews (Hildebrandt and Dityatev, 2015; Varbanov and Dityatev, 2017).

## PolySia Modulates NCAM Functions in Brain and Tumor Development

In brain development, NCAM is implicated in axon guidance and neural cell migration (Maness and Schachner, 2007). Almost all NCAM is polysialylated in the embryonic and perinatal brain, followed by a rapid decrease of polySia and the occurrence of polySia-free NCAM during the early postnatal phase (Oltmann-Norden et al., 2008). As reviewed in great detail elsewhere (Bonfanti, 2006; Nacher et al., 2013), polySia-NCAM is expressed by most neural precursors and immature neurons of the developing brain but restricted to sites of ongoing neurogenesis or plasticity in the adult, including newborn neurons in the neurogenic niches of the subventricular zone and the dentate gyrus, dendrites and axons (mossy fibers) of mature dentate granule neurons, as well as some other cell types, such as subpopulations of interneurons in the cortex and amygdala.

NCAM-negative mice are almost completely devoid of polySia, but show a surprisingly mild phenotype (Cremer et al., 1994). Two prominent defects are the impaired postnatal migration of interneuron precursors from the neurogenic subventricular zone towards the olfactory bulb and the incorrect lamination of hippocampal mossy fibers. Both could be recapitulated by injections of the polySia-degrading enzyme endosialidase into the early postnatal brain indicating that they are caused by the loss of polySia independent of specific NCAM functions (Ono et al., 1994; Seki and Rutishauser, 1998). Although disputed, the most common explanation of these findings is a steric hindrance of cell surface interactions in the presence of polySia-NCAM (for a detailed discussion, see Schnaar et al., 2014). Likewise, and again summarized in more detail elsewhere, the anti-adhesive properties of polySia are considered a major reason for metastatic or invasive growth of polySia-positive tumors such as neuroblastoma, small and non-small cell lung carcinomas or glioblastoma, to name just a few (Hildebrandt et al., 2010; Colley et al., 2014).

Contrasting this rather unspecific mechanistic view, evidence for a specific control of NCAM functions by polySia emerged from studies on neuroblastoma cells and from the analysis of mice with genetic ablation of polysialylation without affecting the expression of NCAM. Both lines of studies were sparked by the identification of the two mammalian polysialyltransferases (polySTs), ST8SIA2 and ST8SIA4, which both are individually able to produce polySia on NCAM (Eckhardt et al., 1995; Kojima et al., 1995; Nakayama et al., 1995; Scheidegger et al., 1995). Although their expression patterns indicate independent transcriptional regulation, the two polySTs often occur simultaneously in tumor cells and during brain development (Hildebrandt et al., 1998a; Hildebrandt et al., 1998b; Ong et al., 1998). In these cases, either both enzymes have to be deleted to obtain polySia-negative cells without affecting NCAM expression, or, as frequently applied to study cellular systems, polySia can be removed by endosialidase treatment.

Bridging tumor cell growth and neuron-like differentiation, polySia-NCAM positive neuroblastoma cells are a particularly interesting model system. PolySia-NCAM levels in serum and on tumors correlate with malignancy of neuroblastoma, but are low after successful therapy (Glüer et al., 1998). Neuroblastoma and most of the human neuroblastoma-derived cell lines use both polySTs to produce polySia (Hildebrandt et al., 1998b; Seidenfaden et al., 2000; Seidenfaden and Hildebrandt, 2001; Valentiner et al., 2011). Based on the initial observation that endosialidase treatment reduces neuroblastoma cell growth, these cells were used to demonstrate that loss of polySia initiates NCAM trans-interactions, leading to reduced proliferation in favor of more robust survival and neuron-like differentiation due to a sustained activation of the ERK signaling pathway (Seidenfaden et al., 2003; Seidenfaden et al., 2006). NCAM and polyST transfections of a polySia- and NCAM-negative neuroblastoma cell line enabled combinatorial interaction studies revealing that polySia is a negative regulator of heterophilic NCAM trans-interactions at cell-cell contacts (Seidenfaden et al., 2003). Further studies in this system identified specific NCAM modules that induce FGF-receptor signaling to activate ERK, or FGF receptor-independent signaling causing reduced motility and enhanced focal adhesion at the cell-substrate interface (Eggers et al., 2011). A receptor for the latter, however, is still elusive.

Notably, the control of heterophilic NCAM trans-interactions by polySia could also be demonstrated for neural precursor cells. Removal of polySia from cell-cell contacts promotes differentiation of subventricular zone-derived neuroblasts, and when exposed to polySia-free NCAM, neuroblasts from wildtype and NCAM knockout mice both respond in the same way by increased formation of neurites and enhanced differentiation towards a calretinin-positive phenotype (Röckle et al., 2008).

Complementary to these cellular studies, the combined analysis of polyST- and NCAM-negative mouse models enabled the dissection of polySia and NCAM functions during mouse brain development. Each of the two polyST knockout lines shows specific phenotypic traits but retains substantial amounts of polySia (Eckhardt et al., 2000; Angata et al., 2004). Their polySia patterns indicate a predominant role of ST8SIA2 during embryonic brain development and in immature neurons of the adult brain, switching towards ST8SIA4-driven polySia synthesis in mature neurons that retain polySia (Eckhardt et al., 2000; Angata et al., 2004; Oltmann-Norden et al., 2008; Nacher et al., 2010). In contrast, *St8sia2*/*St8sia4* double knockout mice are completely devoid of polySia (Weinhold et al., 2005; Angata et al., 2007). These mice recapitulate the major features of NCAM knockout mice that could be phenocopied by endosialidase injections, i.e., impaired migration of olfactory interneuron precursors causing smaller olfactory bulbs, and delamination of mossy fibers (Weinhold et al., 2005; Röckle and Hildebrandt, 2016). In addition, polyST-negative mice show a severe phenotype with postnatal growth retardation, precocious death and major defects in brain development, including a high incidence of progressive hydrocephalus and malformations of major brain axon tracts. All of these severe defects could be rescued by the additional ablation of NCAM in *St8sia2*/*St8sia4/Ncam* triple knockout mice indicating that the regulation of NCAM interactions is a vital developmental function of polySia (Weinhold et al., 2005). This was substantiated by showing that not the loss of polySia per se, but the amount of polySia-negative NCAM present during brain development is directly correlated with the hypoplasia of, e.g., corpus callosum, anterior commissure or internal capsule (Hildebrandt et al., 2009). Combined with the cellular studies described above, the data make a compelling case that the tight regulation of NCAM signaling is a key function of polySia.

## Altered Polysialylation and Neurodevelopmental Predisposition to Psychiatric Disease

Reduced numbers of polySia-positive cells in the hilus region of the hippocampus (Barbeau et al., 1995) and reduced polySia immunoreactivity in the prefrontal cortex (PFC) were detected in schizophrenic patients (Gilabert-Juan et al., 2012). In contrast, polySia in the amygdala was reduced in depressive and increased in bipolar disorder patients (Varea et al., 2012). Furthermore, increased polySia-NCAM levels in the serum of schizophrenic patients have been linked to negative symptoms and cognitive performance. Strikingly, the serum levels were inversely correlated to grey matter reductions in Brodmann area 46 of the left prefrontal cortex, a region implicated in cognitive functions and frequently reported to show early alterations in schizophrenia (Piras et al., 2015).

By genetic studies, variations in *ST8SIA2* have been associated with schizophrenia (Arai et al., 2006; Tao et al., 2007; Gilabert-Juan et al., 2013a; Yang et al., 2015), autism (Anney et al., 2010; Kamien et al., 2014), bipolar disorder (Lee et al., 2011; McAuley et al., 2012; Shaw et al., 2014; Yang et al., 2015) and depression (Kautzky et al., 2015). Arguably, these diseases have common genetic risk factors and neurodevelopmental predispositions creating a possible link between genetic variation of *ST8SIA2* and altered brain development in these disorders.

In schizophrenia, reductions of the internal capsule appear to be linked to thalamocortical dysconnectivity, decreased size of the thalamus, and ventricular enlargement. Based on the correlation of internal capsule hypoplasia with polySia-negative NCAM and severe developmental deficits of thalamocortical connectivity in *St8sia2*/*St8sia4* double knockout mice (Hildebrandt et al., 2009; Schiff et al., 2011), this system was re-evaluated in *St8sia2* and *St8sia4* single knockout mice, together with a comparative behavioral analysis (Kröcher et al., 2015). The neuroanatomical assessment revealed a variable degree of ventricular dilatation as well as size reductions of the thalamus and the internal capsule in *St8sia2*- but not *St8sia4*-deficient mice. This was accompanied by a severely disordered pattern of fibers connecting thalamus and cortex, and reduced glutamatergic thalamic input to the frontal/prefrontal cortex. In a novel object recognition task, both lines showed signs of impaired memory, whereas working memory, prepulse inhibition and amphetamine-induced hyperlocomotion were only affected in the *St8sia2*-deficient mice indicating that compromised brain development caused by the loss of ST8SIA2-dependent polysialylation can lead to schizophrenia-like psychotic behavior (Kröcher et al., 2015). Along the same lines, it has been shown that cognitive deficits of adult *St8sia2*-deficient mice are aggravated by exposure to tetrahydrocannabinol (THC), the main psychoactive compound of cannabis, during adolescence (Tantra et al., 2014). A synergistic negative effect was observed 3 months after the end of THC injections and was accompanied by an imbalance between polySia-positive and -negative NCAM in the hippocampus and altered polySia immunoreactivity in the outer molecular layer of the dentate gyrus. In light of the prominent effects of polySia on synaptic transmission (for reviews, see Hildebrandt and Dityatev, 2015; Varbanov and Dityatev, 2017), altered polysialylation in this synaptic input region of the hippocampus could indeed be a mechanism by which THC acts as an environmental second hit to further disturb a genetically predisposed and neurodevelopmentally vulnerable system. Similarly, in a double hit mouse model of schizophrenia, changes of polySia were detected in hippocampus and PFC together with a marked reduction of the parvalbumin (PV)-positive population of inhibitory interneurons indicating altered excitatory–inhibitory balance in the PFC (Gilabert-Juan et al., 2013b).

PolyST-deficient mice also show severe reductions and altered synaptic connectivity of PV-positive interneurons in the PFC (Kröcher et al., 2014; Curto et al., 2019). This phenotype can be segregated by specific ablation of *St8sia2* in cortical interneurons that are born in the medial ganglionic eminences of the embryonic telencephalon and migrate into the developing cortex (Schuster et al., 2020). PV-positive interneurons are firmly linked to cognitive performance and loss of these cells in the PFC is a frequently reported neuropathological finding in schizophrenia, autism and related disorders (Lewis et al., 2012; Marin, 2012). Therefore, the reduction of PV neurons alone or together with impaired glutamatergic thalamocortical input on inter- and/or projection neurons of the PFC (Bygrave et al., 2016; Bolkan et al., 2017) may contribute to the psychotic behavior of *St8sia2*-deficient mice.

Altered fear behavior, increased aggression, reduced anxiety, and deficits in social interactions are other behavioral traits of *St8sia2*-deficient mice (Angata et al., 2004; Calandreau et al., 2010). As shown recently, local knockdown of *St8sia2* in the early postnatal amygdala was sufficient to reproduce increased aggression and impaired fear learning (Bacq et al., 2020). Both changes in behavior seem to be linked to developmentally impaired glutamatergic synaptic transmission and could be normalized by administration of the partial NMDA-receptor agonist d-cycloserine to the amygdala. In contrast, the local silencing had no effect on anxiety, and the hypoanxiety of *St8sia2* knockout mice could not be normalized by local application, but by ventricular delivery of d-cycloserine. Thus, selected behavioral traits of *St8sia2*-deficient mice could be assigned to developmental alterations in the amygdala.

**FIGURE 2:**
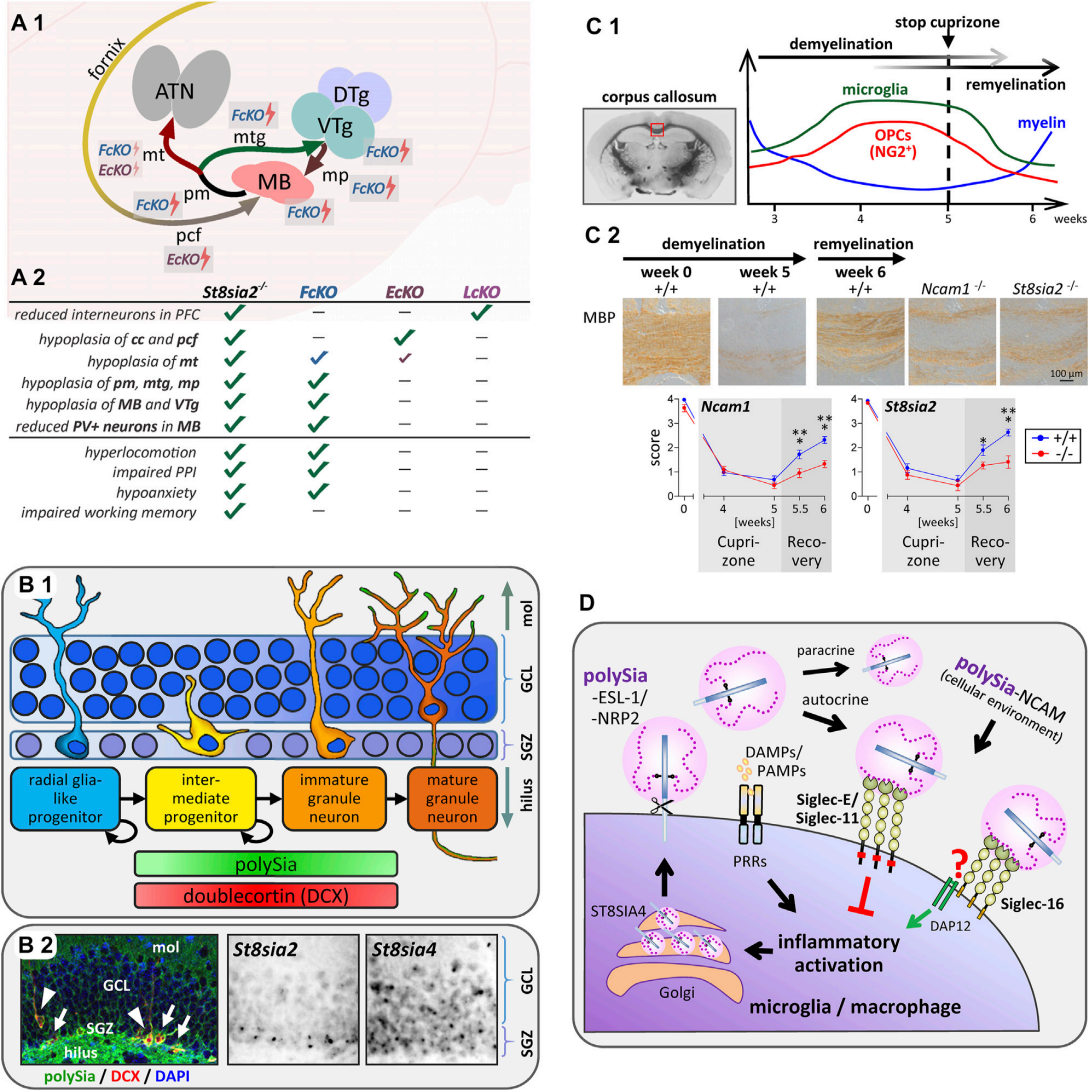
Implications of polysialylation for the neurodevelopmental basis of psychotic behavior, hippocampal neurogenesis, myelin repair and Siglec-mediated immunomodulation. **(A)** Schematic overview of mammillary body (MB) connectivity affected by conventional or conditional knockout of *St8sia2* and its behavioral consequences. **(A1)** The MB is a group of hypothalamic nuclei, which receives its major input from the subiculum of the hippocampus *via* the postcommissural fornix (pcf), and sends projections to the anterior thalamic nuclei (ATN) via the mammillothalamic tract (mt). The mt is formed by collaterals of the principal mammillary tract (pm), which continues as mammillotegmental tract (mtg) towards the ventral and dorsal tegmental nuclei of Gudden in the midbrain (VTg, DTg). Reversely, the mammillary peduncle (mp) projects from these tegmental nuclei to the MB. *St8sia2* ^*−/−*^ mice show hypoplasia of MB and VTg, and of all afferent and efferent MB connections. As indicated by the lightning symbols, both *Emx1-Cre;St8sia2* ^*f/f*^ (*EcKO*) and *Foxb1-Cre;St8sia2* ^*f/f*^ (*FcKO*) show mt hypoplasia, but less pronounced than in *St8sia2* ^*−/−*^ mice, whereas EcKO and *St8sia2* ^*−/−*^ mice show the same hypoplasia of the pcf, and FcKO fully reproduce all other deficits of MB, VTg and their reciprocal connections. **(A2)** Summary of the neurodevelopmental morphological deficits and behavioral traits in *St8sia2* ^*−/−*^ mice that are fully (**✓**), largely (✓), partially (✓), or not (-) reproduced in *FcKO*, *EcKO* or *Lhx6-Cre;St8sia2* ^*f/f*^ (*LcKO*) mice. cc, corpus callosum. Assessed behavioral traits are (i) increased sensitivity of the locomotor response to the psychotropic drug MK-801 (hyperlocomotion), (ii) exacerbated apomorphine-induced impairment of prepulse inhibition of the acoustic startle response (impaired PPI), (iii) reduced anxiety in the elevated plus maze (hypoanxiety), and (iv) impaired spatial working memory in a delayed nonmatch-to-place T-maze task. Data compiled from Küçükerden et al. (2022). **(B)** Scheme of neurogenesis, occurrence of polySia and doublecortin, and expression of polySTs in the dentate gyrus of the hippocampal formation. **(B1)** In the subgranular zone of the dentate gyrus (SGZ), slowly dividing astrocytic, GFAP-positive radial glia-like progenitors give rise to dividing intermediate progenitors, which migrate into the granule cell layer (GCL) while differentiating towards unipolar immature and finally mature granule neurons with polySia-negative somata, but polySia on their mossy fiber axons running through the hilus, as well as on their dendritic arbors in the molecular layer (mol). Stages with doublecortin- and polySia-positive somata are indicated. **(B2)** Coronal mouse brain section with polySia (green) and doublecortin (DCX, red) double-positive intermediate progenitors (arrows) and unipolar immature granule neurons (arrowheads). Nuclear counterstain with DAPI (blue). Detection of polyST mRNAs by *in situ* hybridization indicates expression of *St8sia2* and *St8sia4* in the neurogenic subgranular zone (SGZ) but only *St8sia4* expression persists in the neurons of the granule cell layer (GCL). Immunofluorescent color image reproduced from Tantra et al. Behav Brain Res. 275 (2014), p.173 (Figure 4B) with permission from Elsevier, microscopic greyscale images reproduced from Hildebrandt et al. J Neurochem. 71 (1998), p. 2343 (Fig. 3M,N) with permission from Wiley. **(C)** Remyelination depends on polysialylation of NCAM by ST8SIA2. **(C1)** Course of myelination, oligodendrocyte precursor expansion and microglia activation in the corpus callosum during demyelination induced by cuprizone treatment for 5 weeks followed by remyelination during 1 week after cuprizone withdrawal (based on data reviewed by Skripuletz et al., 2011). **(C2)** Comparison of remyelination in wildtype (+/+), St8sia2−/−, and Ncam−/− mice by immunohistochemical staining and evaluation of myelin basic protein (MBP; reproduced from Werneburg et al., 2017). **(D)** Simplified working model of the proposed feedback regulation of microglia and macrophage activation by polysialylated proteins. Inflammatory activation by pattern recognition receptors (PRRs), responding to damage- and pathogen-associated molecular patterns (DAMPs, PAMPs), leads to ectodomain shedding of polysialylated ESL-1 and NRP2 produced by the polysialyltransferase ST8SIA4 in the Golgi compartment. Shed protein-bound polySia then interacts with murine Siglec-E or human Siglec-11 to inhibit inflammatory activation via signaling through cytoplasmic inhibitory domains (ITIMs, red squares). In humans capable of producing Siglec-16 (see text for details), polySia binding to Siglec-16 can possibly counteract the Siglec-11-mediated inhibition by triggering the association of Siglec-16 with the activating adaptor protein DAP12. PolySia-NCAM in the cellular environment, if present, may exert the same Siglec-mediated effects.

To further dissect neurodevelopmental defects and behavioral consequences of *St8sia2* deficiency, mice with conditional knockout (cKO) of *St8sia2* in cortical interneurons (*Lhx6-Cre;St8sia2* ^*f/f*^) were compared to mice with cKO in the cortical environment (*Emx1-Cre;St8sia2* ^*f/f*^), in the di- and mesencephalon (*Foxb1-Cre;St8sia2* ^*f/f*^), or both (*Foxb1-Cre;Emx1-Cre;St8sia2* ^*f/f*^) (Küçükerden et al., 2022). Unexpectedly, disturbed thalamocortical connectivity could not be observed in any of these cKO lines. However, the same hypoplasia of corpus callosum and fornix was detected in *St8sia2* ^*−/−*^ and *Emx1-Cre* driven cKO mice, while *Foxb1-Cre* driven cKO mice fully reproduced deficits of the mammillary body (MB) and its connectivity, including a prominent reduction of PV-positive mammillary projection neurons and hypoplasia of mammillothalamic and mammillotegmental connections (Figure 2A). Largely consistent with behavioral consequences of MB lesions (Field et al., 1978; Beracochea and Krazem, 1991; Vann, 2009), only mice with these mammillary deficits reproduced a number of psychosis-like symptoms of *St8sia2*-deficient mice (Figure 2A). Linking altered MB connectivity and mental disorders, abnormal neuron densities, reduced numbers of PV neurons, or smaller volumes of the MB have also been observed in schizophrenic, depressive or bipolar patients (Briess et al., 1998; Bernstein et al., 2007). In contrast, only *St8sia2* ^*−/−*^ but none of the cKO mice showed impaired working memory, which therefore seems not to be caused by the mammillary deficits and also not by reductions of cortical interneurons, as these are equally pronounced in *St8sia2* ^*−/−*^ and *Lhx6-Cre;St8sia2* ^*f/f*^ mice. Instead, the working memory deficits may arise from impaired thalamocortical circuits, possibly in combination with altered interneuron functions.

Taken together, the available data in mice and humans support the idea that imbalanced polysialylation can lead to a neurodevelopmental predisposition to psychiatric diseases.

## PolySia and Neurogenesis in Learning and Memory, Aging and Neurodegeneration

Together with doublecortin (DCX), polySia is one of the most frequently used markers to identify intermediate progenitor stages during adult neurogenesis (Lledo et al., 2006; Kempermann et al., 2018) (Figure 2B). Concerning polySia functions in this context, *in vitro* findings on its role in regulating differentiation of subventricular zone-derived neuroblasts (Röckle et al., 2008; see section 2) received support by a concomitant study describing that enzymatic polySia removal leads to less migration in favor of enhanced maturation of progenitors in the neurogenic subgranular zone (SGZ) of the dentate gyrus *in vivo* (Burgess et al., 2008). A related finding is the increase of DCX-positive progenitors in the SGZ of *St8sia4*-deficient mice (Nacher et al., 2010). More recently, it could be demonstrated that memory deficits of *St8sia4* knockout mice in a novel object recognition task could be overcome by environmental enrichment (Zerwas et al., 2016). *St8sia4* deficiency had no effect on neurogenesis per se, but environmental enrichment was associated with an increased number of polySia-positive cells in the SGZ, i.e., of early progenitors with an ST8SIA2-dependent polySia production. Possibly, a larger pool of newborn polySia-positive progenitors supports the beneficial effects of enhanced neurogenesis on memory performance by mice housed in an enriched environment.

In light of these findings, it is intriguing that a recent study on human aging found no age-related decline of hippocampal neurogenesis, but a clear correlation of age with a decrease in the number of polySia-positive, unipolar immature granule cells in the SGZ and the adjacent granule cell layer, which was interpreted as an indicator of reduced neuroplasticity (Boldrini et al., 2018). Furthermore, an early decline in the percentage of DCX-positive cells that express polySia has been reported during progression of Alzheimer’s disease (Moreno-Jimenez et al., 2019). In contrast, this specific cell population was increased in Huntington’s disease and not altered in patients with frontotemporal dementia, alpha-synucleinopathies (Parkinson’s disease and dementia with Lewy bodies), or amyloid lateral sclerosis, each of which, however, displayed specific alterations of other cellular features in the dentate gyrus (Terreros-Roncal et al., 2021).

## PolySia in Myelin Maintenance and Repair

In the brain, the insulating sheath of myelin around axons is formed by oligodendrocytes, which are generated from oligodendrocyte precursor cells (OPCs). PolySia levels decrease during OPC differentiation and myelination (Trotter et al., 1989; Nait Oumesmar et al., 1995). *In vitro* data indicate a dual role of polySia in promoting OPC chemotaxis but inhibiting myelin formation (Charles et al., 2000; Zhang et al., 2004). Correspondingly, in multiple sclerosis lesions polySia has been detected on OPCs and on chronically demyelinated axons (Charles et al., 2002; Nait-Oumesmar et al., 2007). However, it seems that the downregulation of polySia during OPC differentiation is a major prerequisite for efficient myelin formation, because mice with forced expression of *St8sia4* in the oligodendrocyte lineage, but not in neurons, displayed a reduced myelin content and formed less compact myelin (Fewou et al., 2007; Bakhti et al., 2013; Fewou et al., 2019). *St8sia2*-deficient mice show axonal damage and aberrant myelin maintenance linked to deficits in oligodendrocyte development, possibly caused by altered PDGF receptor signaling (Szewczyk et al., 2017). A dual role of polySia for myelin repair has been derived from analyses of remyelination after cuprizone-induced demyelination. Remyelination was slightly accelerated in the absence of ST8SIA4, but equally impaired in *St8sia2*- and in *Ncam*-deficient mice (Koutsoudaki et al., 2010; Werneburg et al., 2017) (Figure 2C). These seemingly discrepant findings might be explained by a cell autonomous impairment of OPC differentiation in the absence of ST8SIA2 or NCAM, but premature differentiation in ST8SIA4-negative cultures. This opposing role of the two polySTs is supported by their sequential expression during OPC differentiation. The importance of polySia regulation is reinforced by showing that retinoic acid, a potent promoter of OPC differentiation and remyelination (Huang et al., 2011), enhances *St8sia2* expression and that artificial polysialylation of the cell surface accelerates OPC differentiation (Werneburg et al., 2017).

Together, the data identify polysialylation and polySTs as promising therapeutic targets to support myelin repair in demyelinating diseases.

## PolySia in Microglia and Macrophage Activation

Following a first description of neuroprotective effects by polySia interactions with microglia transduced with the human-specific inhibitory immunoreceptor Siglec-11 (Wang and Neumann, 2010), the potent inhibition of inflammatory microglia and macrophage activation by soluble, free or protein-bound polySia was demonstrated in murine microglia and human THP-1 macrophages (Shahraz et al., 2015; Werneburg et al., 2015a; Kallolimath et al., 2016; Werneburg et al., 2016). Concomitantly, NRP2 and ESL-1 (gene name *Glg1*) were identified as polySia carriers in cultured microglia and THP-1 macrophages as well as in injury-induced microglia in brain slice cultures (Werneburg et al., 2015a; Werneburg et al., 2016). Remarkably, the two polysialylated proteins accumulated in the Golgi compartment, but in response to LPS-induced inflammatory activation they rapidly were translocated to the cell surface and released by ectodomain shedding (see Figure 1C). Based on the effect of soluble polySia, it was assumed that polySia on the released proteins is involved in negative feedback regulation of microglia activation. This could be confirmed by identifying Siglec-E as the receptor responsible for polySia-mediated inhibition in murine microglia (Thiesler et al., 2021) (Figure 2D). This study also revealed that the shedding of polysialylated proteins continues for at least 24 h after LPS-induction and provided first evidence for accumulation and shedding of polySia by injury-induced microglia *in vivo*.

A first indication of the potential for therapeutic application of soluble polySia came from its beneficial effects on laser-induced retina damage in transgenic mice expressing human Siglec-11 in microglia and macrophages (Karlstetter et al., 2017). Although the interpretation might be hampered by concurrent effects of transgenic Siglec-11 and intrinsic murine Siglec-E, this pioneer study demonstrated a potent reduction of injury–induced microglia/macrophage activation and complement deposition. With regard to any therapeutic application of polySia, it also has to be considered that humans have a second polySia-binding immunoreceptor, Siglec-16, with essentially the same extracellular domain as Siglec-11, but activating immune signaling, which may have evolved to balance responses to polySia-presenting pathogens (Cao et al., 2008; Schwarz et al., 2017). However, the majority of the human population is homozygous for an inactive pseudogene, *SIGLEC16P*, with a four base pair deletion disrupting the open reading frame, and, therefore, not able to express functional Siglec-16 (Hayakawa et al., 2005; Wang et al., 2012; Hayakawa et al., 2017). So far, virtually nothing is known about the role of Siglec-16 in the brain, in part because there is no known counterpart of Siglec-16 in non-primates.

## Conclusion and Perspectives

Despite increasing evidence for specific polySia interactions, the modulation of NCAM is still the major developmental function of polySia. A neglected topic of this review is the long history of proposed interactions of polySia with BDNF and other growth factors or chemokines, mainly because their relevance in the brain is largely speculative (comprehensively reviewed by Colley et al., 2014; Schnaar et al., 2014). The recently described cell autonomous role of polySia in the migration of cortical interneuron progenitors might be based on binding of BDNF or chemokines (Schuster et al., 2020). Hence, this system would be suited to test, e.g., for similarities to the mode of polySia interactions with the chemokine CCL21 in chemotactic migration of dendritic cells (Kiermaier et al., 2016). Quite a few intriguing questions relate to the role(s) of the two different polySia-carriers shed by activated microglia/macrophages and the relevance of the polySia-Siglec axis for innate immune responses in the brain, ranging from injury-induced activation to modulation of inflammatory processes in aging, neurodegeneration, or demyelinating disease, and their future exploration may lead to new therapeutic avenues.
